# Long-term disease control of monomorphic epitheliotrophic intestinal T-cell lymphoma (MEITL) with CNS involvement in a caucasian patient by allogeneic hematopoietic cell transplantation

**DOI:** 10.1038/s41409-025-02747-3

**Published:** 2025-11-15

**Authors:** Markus Lindauer, Florian Reissfelder, Ute Hegenbart, Thomas Luft, Uwe M. Martens, Peter Dreger

**Affiliations:** 1 Department Medicine III, SLK Clinics Heilbronn GmbH, Heilbronn, Germany; 2 Institute of Pathology, SLK Clinics Heilbronn GmbH, Heilbronn, Germany; 3 Department Medicine V, University of Heidelberg, Heidelberg, Germany

**Keywords:** Stem-cell research, Stem-cell therapies

## Introduction

Monomorphic epitheliothrophic intestinal T-cell lymphoma (MEITL), formerly known as type II enteropathic T-cell lymphoma, is an aggressive form of peripheral T-cell lymphoma (PTCL) primarily originating from intraepithelial lymphocytes of the intestine [1]. Arising mostly in the small intestine with solitary or multifocal, often destructing lesions, it can disseminate in lymph nodes and non-intestinal organs including bone marrow and the central nervous system (CNS) [1–4]. It predominantly affects Asian and Hispanic populations but very rarely also Caucasians [1, 5]. Generally, MEITL responds poorly to anthracycline-based lymphoma-typic induction chemotherapy, prompting investigators to explore more aggressive first-line treatment including high-dose therapy with autologous hematapoietic cell transplantation (auto-HCT). Although this strategy may be associated with better disease control, almost all patients will eventually relapse [6, 7]. Accordingly, the prognosis of MEITL is dismal with median survival times around 12 months and only few long-term survivors [2, 3, 6, 7]. The outcome is particularly poor if CNS involvement is present, a recent literature review identified only a single survivor among 12 cases reported [4]. Thus, expert recommendations advocate consolidation of MEITL responses by cellular immunotherapy through allogeneic hematopoietic cell transplantation (alloHCT) in analogy to other aggressive extranodal PTCL with similar biological behavior, such as hepatosplenic T-cell lymphoma [8, 9] and acute/lymphoma-type adult T-cell leukemia/lymphoma [10, 11], although the evidence basis for this is very poor.

Here we report a case of MEITL affecting a caucasian patient that could be durably controlled by alloHCT despite prior relapse in the CNS. The patient gave full informed consent to all interventions described and the use of her clinical data for scientific purposes.

## Clinical case

**Fig. 1:**
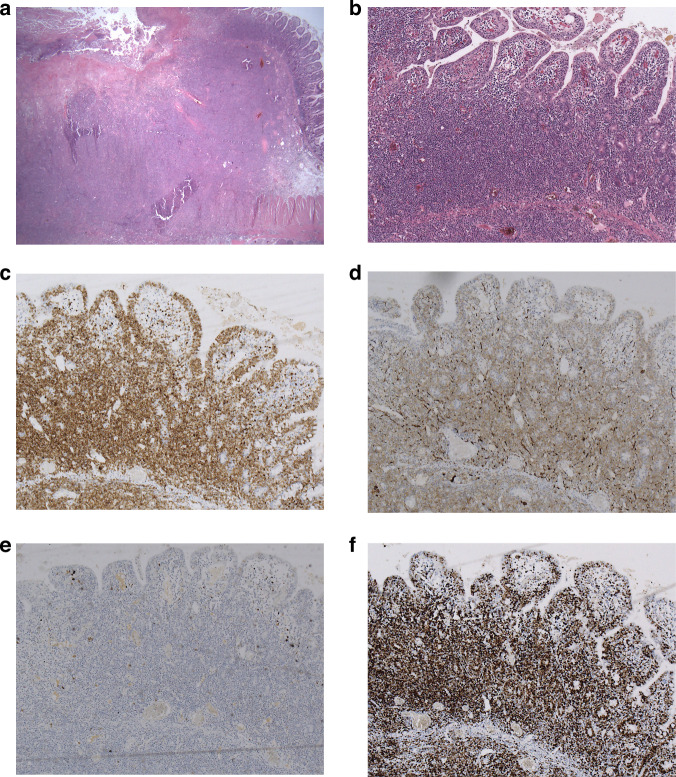
Histopathological work-up of tumor tissue at diagnosis. Low-power view showing the transmural atypical lymphoid infiltrate in the small bowel (**a**). High-power view highlighting the intraepithelial neoplastic T-cells (**b**). Immunhistochemical analyses showing expression of CD8 (**c**) and CD56 (**d**) but negativity for CD30 (**e**), and a high KI-67 proliferation index (**f**).

A 65-year-old female Caucasian patient underwent segmental small bowel resection because of an ileus caused by a stenosing tumor of the ileum. There was no history of inflammatory bowel disease. Histopathological work-up disclosed a monomorphic tumor of 45 mm diameter originating from the epithelium with transmural infiltration of the bowel wall. It consisted of actively proliferating medium-sized lymphoid cells (Ki-67 > 90%) expressing CD3, CD8, CD56, and bcl2 while being negative for CD4, CD5, CD30, all B-cell markers, ALK, bcl6, cyclin-D1, and EBV; consistent with a diagnosis of MEITL (Fig. 1). Staging with complete imaging including brain MRI and bone marrow biopsy yielded no extra-intestinal involvement, but additional jejunal lesions were identified on re-laparotomy, compatible with an Ann Arbor stage IVA [12]. The patient achieved complete response on 6 cycles of CHOP but refused to undergo consolidative autoHCT. 3 months after completion of induction chemotherapy, she presented with tetraparesis, due to isolated meningeosis based on presence of malignant T-cells in the CSF expressing the MEITL immunophenotype. Intrathecal and systemic chemotherapy with high-dose methotrexate/ara-c led to resolution of tetraparesis and CSF clearance, but MRI imaging after 3 cycles of methotrexta/ara-c upon neurological deterioration revealed a new intraspinal tumor at L2/3. Local fractionated radiotherapy (13x3Gy) was instituted and resulted in complete resolution of the spinal tumor.

## Results and discussion

In the absence of other MEITL manifestations the patient underwent myeloablative conditioning with thiotepa, busulfan, fludarabine, and ATG and received a peripheral blood-derived hematopoietic cell allograft from a 10/10-matched male unrelated donor, using tacrolimus and MMF for GVHD prophylaxis. Apart from a brief steroid-sensitive episode of grade-I acute skin GVHD, the early post-transplant course was uneventful, and tacrolimus could be tapered off from day +90 onwards. However, six moths post alloHCT after almost complete withdrawal of systemic immunosuppression, tacrolimus needed to be re-escalated because of moderate cutaneous and mucosal chronic GVHD along with eosinophilia, leading to prompt resolution of clinical and laboratory signs of chronic GVHD. Comprehensive diagnostic work-up 10 months after alloHCT documented full donor chimerism and the complete absence of lesions suspicious for lymphoma persistence. The further course was uncomplicated, and at the latest visit 57 months after transplantation the patient presented as a full donor chimera without clinical or laboratory signs for MEITL persistence and with completely normalized hematopoiesis and immune status.

There is barely information on alloHCT for MEITL published to date. Ishibashi et al. described a patient who underwent alloHCT after partial response to induction chemotherapy but died 9 months after diagnosis without providing further details [2]. Recently, Min et al. reported a series of 35 newly diagnosed MEITL cases of whom 10 achieved complete response after first-line chemotherapy [3]. Of these, 7 patients proceeded to transplantation. Whilst all four patients undergoing autoHCT relapsed and died, all three patients receiveing alloHCT remained alive and disease-free at a median follow-up of 33 months. Six additional patients underwent alloHCT in the salvage setting, with two long-term survivors, and two relapse and toxic deaths each. Four of the nine allografted patients had moderate chronic GVHD. Only 7 of the total 35 patients (20%) had an ongoing complete remission at their latest follow-up. Of these, five were alloHCT recipients and one patient who had been consolidated with autoHCT plus cytokine-induced killer cells within a trial [3].

While the Min study convincingly demonstrated feasibility and efficacy of alloHCT in MEITL, our case extends this evidence by showing for the first time that allotransplantation can be effective also in non-Asian patients and in those who had CNS involvement with MEITL. Although still speculative and limited by the very small case numbers published to date, the striking superiority of alloHCT over autoHCT and the history of chronic GVHD in many of the long-term survivors after allogeneic transplantation are strongly suggestive of clinically meaningful graft-versus-lymphoma activity as the main driver of long-term disease control associated with alloHCT in MEITL. Anyhow, our case supports expert consensus recommending early consideration of alloHCT in the treatment algorithm of this devastating malignancy [8].

## Data Availability

The datasets generated during and/or analyzed during the current study are available from the corresponding author on reasonable request.
